# Knowledge of Early Stroke Detection Among Patients With Hypertension in Urban Population: A Cross‐Sectional Survey

**DOI:** 10.1155/nri/5012364

**Published:** 2026-11-01

**Authors:** Rosyidah Arafat, Alif Muhammad Islam, Elly L. Sjattar, Syahrul Ningrat, Lee Wang Ling

**Affiliations:** ^1^ Faculty of Nursing Hasanuddin University Makassar Indonesia unhas.ac.id; ^2^ Nursing Departement Faculty of Medicine University of Malaya Kuala Lumpur Malaysia um.edu.my

**Keywords:** cross-sectional, early stroke detection, hypertension, knowledge

## Abstract

**Background:**

Hypertension is one of the primary risk factors for stroke, and nearly 80% of stroke cases can potentially be prevented through increased awareness and early detection. However, previous studies indicate that patients with hypertension often demonstrate inadequate stroke prevention behaviors. Therefore, this study aims to assess knowledge related to early stroke detection among hypertensive patients.

**Methods:**

This descriptive quantitative study utilized a cross‐sectional design, with 236 respondents recruited through consecutive sampling. Data were collected using the Stroke Recognition Questionnaire (SRQ) and analyzed through univariate statistical methods, including frequency, percentage, mean, and standard deviation.

**Results:**

Among the 236 respondents, 53.4% demonstrated a moderate level of knowledge, 31.4% had low knowledge, and the remaining respondents showed a high level of knowledge. The average knowledge score was 64.61 (SD = 13.57; range = 26–97). Participants aged 20–44 years had the highest proportion of high knowledge levels (11.4%), whereas older adults aged over 60 years were more commonly classified in the low‐knowledge category (36.1%).

**Conclusion:**

The greater proportion of low knowledge among older adults highlights a priority population for targeted education. Integrating structured stroke recognition education into routine hypertension care may strengthen preparedness for early symptom recognition and facilitate timely access to emergency stroke services.

## 1. Introduction

Hypertension is a leading cause of cardiovascular disease and premature mortality worldwide, with its prevalence increasing particularly in low‐ and middle‐income countries [1]. Hypertension affects approximately one in three adults globally, and its most common and severe complication is stroke [2]. Previous research conducted in Indonesia indicated that most individuals with hypertension exhibited insufficient stroke prevention behaviors. A key factor associated with these poor preventive practices was limited awareness and understanding of stroke [3]. An increased level of public awareness about stroke plays a crucial role in enhancing stroke prevention strategies, especially within high‐risk populations. Nearly 80% of first‐time stroke incidents may be prevented by improving knowledge and understanding of stroke risk factors [4]. Generally, people seek medical care only after experiencing severe clinical symptoms associated with substantial functional impairment, whereas mild symptoms are frequently overlooked. Nevertheless, untreated mild symptoms can also result in disability and death if appropriate medical intervention is not provided promptly [5].

Insufficient knowledge regarding stroke risk factors, symptoms, and early management may lead individuals to delay recognizing stroke as a medical emergency requiring immediate treatment. Consequently, this may result in delays in seeking medical assistance [6]. A study conducted by Upoyo et al. (2021) demonstrated that the majority (65.7%) of patients with hypertension exhibited poor stroke prevention behaviors. Many patients were unaware that hypertension is a risk factor for stroke, leading them to neglect stroke prevention practices [3]. Therefore, this study aims to identify the level of knowledge regarding early stroke prevention among hypertensive patients living in urban areas.

## 2. Methods

### 2.1. Design and Sample

This research employed a cross‐sectional design involving 236 participants. The sample size was calculated using the Slovin formula with a 5% error tolerance. A nonprobability sampling approach was applied, specifically consecutive sampling, in which respondents who satisfied the inclusion criteria were enrolled sequentially. Participants eligible for the study were individuals diagnosed with hypertension who agreed to take part in the research.

### 2.2. Instruments

The level of knowledge was measured using the Indonesian version of the Stroke Recognition Questionnaire (SRQ), which had undergone validity testing for all instrument items based on the original SRQ. Both statements related to stroke symptoms and nonstroke symptoms, as well as stroke risk factors and nonrisk factors, were found to be valid, with each item showing an r‐value higher than the r‐table value of 0.361. The questionnaire consisted of two main sections: 17 items regarding stroke symptoms (10 positive statements and 7 negative statements) and 18 items concerning stroke risk factors [7]. Knowledge levels were classified into three categories: low, moderate, and high. According to the Modified Bloom’s cut‐off criteria, scores below 60% were categorized as poor knowledge, scores between 60% and 79% as moderate knowledge, and scores between 80% and 100% as high or sufficient knowledge [8, 9].

### 2.3. Data Collection Procedure

Data were collected in the primary clinic. The collection began after obtaining ethical approval. Potential respondents were identified according to the inclusion criteria. Those who agreed signed informed consent and completed the questionnaire themselves, with assistance if needed. Answers were checked immediately after completion.

### 2.4. Data Analysis

This research involved both numerical and categorical data. Numerical variables, including age and duration of hypertension, were summarized using descriptive statistics such as the mean, standard deviation, and minimum and maximum values. In contrast, categorical variables such as sex, educational attainment, occupation, marital status, and knowledge level regarding early stroke detection were analyzed through frequency distribution tables and presented in terms of frequencies and percentages for each category.

## 3. Results

The findings on respondents’ knowledge of early stroke detection indicated that, among the 236 participants, most respondents were classified in the moderate knowledge category, totaling 126 individuals (53.4%), while 74 respondents (31.4%) were categorized as having low knowledge (Table 1). In general, knowledge scores ranged between 26 and 97, with an average score of 64.61 and a standard deviation of 13.57.

**TABLE 1:** Respondents’ knowledge about early stroke detection.

Categories	*N* = 236	%	Mean ± SD	Min	Max
Score < 59 (low)	74	31.4			
Score 60–79 (moderate)	126	53.4	64.61 ± 13.57	26	97
Score > 80 (high)	36	15.3			

Table 2 presents the distribution of respondents’ knowledge levels regarding early stroke detection according to their demographic and clinical characteristics. Based on age, respondents aged ≥ 60 years constituted the largest proportion with moderate knowledge (24.2%), followed by those aged 45–59 years (22.9%). High knowledge levels were relatively low across all age groups, with the highest proportion observed among respondents aged 45–59 years (5.5%).

**TABLE 2:** Knowledge of early stroke detection based on respondents’ characteristics.

Respondents’ characteristic	Low		Moderate		High	
	*n*	%	*n*	%	*n*	%
Age						
10–19 years	—	—	1	0.4	—	—
20–44 years	8	3.4	14	5.9	11	4.7
45–59 years	27	11.4	54	22.9	13	5.5
≥ 60 years	39	16.5	57	24.2	12	5.1
Gender						
Male	21	8.9	36	15.3	15	6.4
Female	53	22.5	90	38.1	21	8.9
Marital status						
Married	55	23.3	92	39.0	34	14.4
Widowed/unmarried	19	8.1	34	14.4	2	0.8
Education Level						
Elementary school	33	14	36	15.3	5	2.1
High school	39	16.5	71	30.1	12	5.1
Graduate degree	2	0.8	19	8.1	19	8.1
Employment status						
Working	19	8.0	31	13.1	20	8.5
Retired and unemployed	55	23.3	95	40.3	16	6.8
Duration of hypertension						
< 5 years	42	17.8	71	30.1	25	10.6
≥ 5 years	32	13.6	55	23.3	11	4.7

Regarding gender, female respondents showed higher proportions in all knowledge categories compared to males. Most females had moderate knowledge (38.1%), while only 8.9% demonstrated high knowledge. Among males, 15.3% had moderate knowledge and 6.4% had high knowledge. In terms of marital status, married respondents represented the majority of participants with moderate knowledge (39.0%) and high knowledge (14.4%). Conversely, widowed or unmarried respondents had lower proportions of high knowledge (0.8%).

Based on educational level, respondents with a high school education accounted for the largest proportion with moderate knowledge (30.1%) and low knowledge (16.5%). Participants with a graduate degree demonstrated comparatively better knowledge, with an equal proportion categorized as moderate and high knowledge (8.1% each).

Regarding employment status, retired and unemployed respondents mostly had moderate knowledge (40.3%) and low knowledge (23.3%). According to the duration of hypertension, respondents with hypertension for less than 5 years mostly had moderate knowledge (30.1%) and also showed the highest proportion of high knowledge (10.6%). Meanwhile, respondents with hypertension for ≥ 5 years had lower proportions of high knowledge (4.7%). In summary, moderate knowledge regarding early stroke detection predominated among respondents. Higher educational attainment and employment appeared to be associated with better knowledge levels.

Table 3 shows that the respondents generally demonstrated good recognition of classic stroke symptoms, particularly motor and balance‐related signs. However, knowledge gaps remained regarding less common stroke manifestations such as confusion and double vision, and misconceptions about several nonstroke symptoms were still evident.

**TABLE 3:** Respondents’ knowledge about stroke symptoms.

Items	Yes (%)	No (%)
Stroke symptoms		
Confusion	92 (38.98)	144 (61.02)
Double vision	80 (33.90)	156 (66.10)
Weakness of one side of body	182 (77.12)	54 (22.88)
Numbness on one side of face	181 (76.69)	55 (23.31)
Loss of balance	193 (81.78)	43 (18.22)
Trouble walking	185 (78.39)	51 (21.61)
Sudden severe headache	125 (52.97)	111 (47.03)
Slurred or garbled speech	159 (67.37)	77 (32.63)
Sudden unexplained dizziness	133 (56.36)	103 (43.64)
Trouble with coordination	173 (73.31)	63 (26.69)
Nonstroke symptoms		
Difficulty breathing	39 (16.53)	197 (83.47)
Chest pain	68 (28.81)	168 (71.19)
Swollen ankles	101 (42.80)	135 (57.20)
Heartburns	69 (29.24)	167 (70.76)
Fever	57 (24.15)	179 (75.85)
Leg cramps	145 (61.44)	91 (38.56)
Extreme tiredness	112 (47.46)	124 (52.54)

In Table 4, respondents demonstrated good understanding of major clinical stroke risk factors such as hypertension and hyperlipidemia. Nevertheless, knowledge gaps remain regarding lifestyle‐related risks and several nonmedical factors, indicating the need for improved health education on stroke prevention.

**TABLE 4:** Respondents’ knowledge about stroke risk factors.

Items	Yes (%)	No (%)
Stroke risk factors		
Alcohol use (> 2 drinks per day)	99 (41.95)	137 (58.05)
Irregular heartbeat	123 (52.12)	113 (47.88)
High blood pressure	189 (80.08)	47 (19.92)
Diabetes mellitus	141 (59.75)	95 (40.25)
High blood cholesterol	166 (70.34)	70 (29.66)
Lack of physical activity	149 (63.14)	87 (36.86)
More than 20 pounds overweight	111 (47.03)	125 (52.97)
History of having a heart attack	109 (46.19)	127 (53.81)
Smoking cigarettes	123 (52.12)	113 (47.88)
History of neck vein disease	120 (50.85)	116 (49.15)
Nonstroke risk factor		
Lyme disease	25 (10.59)	211 (89.41)
Iron deficiency	76 (32.20)	160 (67.80)
Alzheimer disease	57 (24.15)	179 (75.85)
Travel to foreign countries	26 (11.02)	210 (88.98)
Low levels of calcium in diet	80 (33.90)	156 (66.10)
Varicose veins	106 (44.92)	130 (55.08)
Living close to a power plant	44 (18.64)	192 (81.36)
Exposure to too much sunlight	33 (13.98)	203 (86.02)

## 4. Discussion

People with hypertension are approximately four times more likely to experience a stroke compared to individuals with normal blood pressure [10]; therefore, recognizing the risk factors and early signs of stroke is essential for effective hypertension management and stroke prevention [11]. The current study found that 53.4% of participants had a moderate level of knowledge about stroke, which is consistent with previous research conducted in Ethiopia showing that approximately 40% of participants had moderate awareness of stroke symptoms [12]. These studies underscore the widespread gap in stroke‐related knowledge and highlight the importance of targeted educational programs to enhance understanding, especially in identifying risk factors and early warning signs to support early and timely intervention.

The study reveals varying levels of knowledge across different stroke‐related domains, with the highest awareness in warning signs and symptoms, while awareness of risk factors and diagnostic tests was relatively low. These findings align with the other study, which reported that 70% of participants recognized stroke symptoms, but only 35% were aware of its risk factors. Both studies highlight a consistent gap in knowledge regarding stroke risk factors and diagnostic approaches, underscoring the need for comprehensive educational programs to improve awareness across all domains, particularly in understanding stroke causes and early detection methods [13].

The present study examines the personal characteristics linked to the level of knowledge regarding early stroke detection among hypertensive patients. Factors affecting awareness included age, education, and occupation. In this study, it was found that participants with a high school education and a graduate degree had better knowledge about stroke, and that literate individuals had more protective attitudes and adopted positive attitudes toward stroke prevention [14]. Participants under 60 years of age had better knowledge. This finding may be attributed to age‐related declines in sensory and cognitive functions [15], which can reduce the ability to understand and process health information. In addition, older adults frequently encounter greater communication challenges compared to younger individuals, including difficulty understanding explanations from healthcare providers or written medical instructions. These barriers may contribute to inappropriate healthcare use or poor health management. Therefore, improving health literacy among older adults is essential for supporting disease prevention and effective health management, as it enables them to better understand their health condition and related health information [16, 17]. Employed individuals demonstrated better knowledge about stroke than unemployed individuals, which may be related to having a stable income. Previous studies have also reported that people with higher socioeconomic status are more likely to engage in effective stroke prevention practices because they have greater access to resources, resulting in better preventive behaviors compared to those with lower income levels [18–20].

## 5. Conclusion

Most patients with hypertension demonstrated only moderate knowledge of early stroke recognition, while nearly one‐third had low knowledge. The greater proportion of low knowledge among older adults highlights a priority population for targeted education. Integrating structured stroke recognition education into routine hypertension care may strengthen preparedness for early symptom recognition and facilitate timely access to emergency stroke services.

## Author Contributions

Rosyidah Arafat and Alif Muhammad Islam: conceptualization, resources, data curation, funding acquisition, validation, investigation, visualization, methodology, and project administration,

Elly L. Sjattar and Syahrul Ningrat: software, formal analysis, and writing–original draft.

Lee Wang Ling: supervision and writing–review and editing.

## Funding

This study was supported in part by grants from the Research and Community Service Institute of Hasanuddin University (Grant nos. 01939/UN4.1.7/PT.01.03/2026).

## Ethics Statement

Ethical approval permission was submitted and approved by the Ethics Committee of the Faculty of Nursing, Hasanuddin University, with approval letter number: 430/UN4.18.3/TP.01.02/2025. All eligible participants were informed of the aims and procedures of the study, the benefits and potential risks, the time required for the study, their rights to privacy, confidentiality, and the ability to withdraw from the study without losing healthcare service benefits. All participants signed a consent form before the research started.

## Consent

Please see the Ethics Statement.

## Conflicts of Interest

The authors declare no conflicts of interest.

## Data Availability

Data are available on request from the corresponding author.
